# Ramadan during pregnancy and birth weight of newborns

**DOI:** 10.1017/jns.2017.70

**Published:** 2018-02-01

**Authors:** Ary I. Savitri, Dwirani Amelia, Rebecca C. Painter, Mohammad Baharuddin, Tessa J. Roseboom, Diederick E. Grobbee, Cuno S. P. M. Uiterwaal

**Affiliations:** 1 Julius Center for Health Sciences and Primary Care, University Medical Center Utrecht, Utrecht, The Netherlands; 2 Budi Kemuliaan Hospital, Jl. Budi Kemuliaan No. 25, Jakarta Pusat 10110, Indonesia; 3 Department of Obstetrics and Gynecology, Academic Medical Center, Meibergdreef 9, 1105 AZ Amsterdam, The Netherlands; 4 Department of Clinical Epidemiology, Biostatistics and Bioinformatics, Academic Medical Center, Meibergdreef 9, 1105 AZ Amsterdam, The Netherlands

**Keywords:** Ramadan, Fasting, Pregnancy, Birth weight

## Abstract

Previous studies suggest that Ramadan exposure during pregnancy might affect the health of women and their babies, particularly through the effect of fasting. This study aimed to evaluate the association between Ramadan exposure and fasting during pregnancy on the birth weight of newborns. This study concerned 1351 pregnant women from a prospective cohort in Jakarta, Indonesia. Ramadan exposure was based on the actual overlap between Ramadan and pregnancy. Women's fasting behaviour was recorded among 139 women who came for antenatal care between 10 July 2013 and 7 August 2013, and those who had fasted for at least 1 d (*n* 110) were classified as exposed to Ramadan fasting. Furthermore, a 24 h dietary recall was performed and repeated 1 month later. Birth weight of newborns who were exposed to Ramadan during pregnancy did not significantly differ from those who were not, both in the total and trimester-specific analysis. Maternal fasting did not seem to affect the birth weight of newborns (−72 (95 % CI −258, 114) g; *P* = 0·44), although there was a non-significant trend towards lower birth weight with fasting in the second and third trimester. Women who fasted had significantly lower total energy, macronutrient and water intake as compared with women who did not. Women's intake was also lower during Ramadan (regardless of their fasting behaviour) as compared with 1 month later. Lifestyle changes that occur with Ramadan and fasting during pregnancy are associated with lower reported energy intake. We cannot conclude on the effect of fasting on birth weight due to low statistical power.

An adverse fetal environment could have serious consequences on health outcomes in offspring. Exposures such as suboptimal maternal diet, smoking and stress could inhibit fetal growth and development, which further cause lower birth weight and other poor birth outcomes^(^^1^^–^^3^^)^. Studies on the long-term impact of fetal growth restriction, as represented by lighter weight at birth, showed a greater risk of later chronic diseases, including CHD, stroke, type 2 diabetes, hypertension, and other cognitive and emotional problems^(^^4^^–^^8^^)^.

During the month of Ramadan when daytime fasting is obligated to every adult Muslim, various degrees of behavioural changes may occur among pregnant Muslim women. Meal frequencies are usually reduced to two times per d; one large meal when breaking the fast in the evening and another smaller meal during *sahoor* at dawn. Many seasonal meals which consist of sugary and fatty foods are commonly served during this month^(^^9^^–^^13^^)^. Physical activities and sleeping pattern are also affected, as people tend to be more active at night. Ramadan lasts for 29–30 d and shifts forward by approximately 11 d each year, since it is based on the Islamic lunar calendar^(^^11^^–^^14^^)^.

Three out of every four pregnant Muslim women are exposed to Ramadan^(^^15^^)^. According to the Islamic rule, pregnant women, together with breastfeeding mothers, are exempted from the obligation to fast during Ramadan. They are permitted to postpone their fasting until after delivery or to feed one poor person for each day they do not fast. However, many women choose to fast during pregnancy to share the spiritual experience with their family. The proportion of fasting pregnant Muslim women varies globally, ranging from 50–70 % (in Iran, The Netherlands)^(^^16^^–^^18^^)^ to 70–90 % (in England, Singapore, USA, Gambia and Yemen)^(^^15^^,^^19^^–^^21^^)^.

Despite its widespread adherence, evidence on the health effects of Ramadan fasting during pregnancy (both maternal and fetal) is still limited. Dietary intake and weight gain of fasting pregnant women were reported to be less than in the non-fasting^(^^22^^,^^23^^)^. Metabolic alterations associated with fasting have also been reported, which include reduction in serum glucose and insulin levels^(^^24^^)^ and elevation in serum TAG, cortisol and leptin concentrations^(^^25^^,^^26^^)^. Several studies showed no association between Ramadan exposure during pregnancies with birth weight, or the risk of low birth weight^(^^16^^–^^18^^,^^22^^,^^26^^–^^31^^)^. On the contrary, several other studies reported a lower birth weight with Ramadan exposure^(^^32^^,^^33^^)^. Many of these studies, however, did not differentiate Ramadan exposure (as overlap between pregnancy and the month of Ramadan) from actual fasting exposure, which potentially leads to dilution of effects.

In the present study, we used data from a large prospective cohort of Indonesian Muslim pregnant women to evaluate both the effect of Ramadan exposure during pregnancy and maternal fasting on the birth weight of newborns. Maternal dietary intakes were evaluated during and after Ramadan, both in the fasting and non-fasting women, in an attempt to seek for possible explanatory factors.

## Materials and methods

### Study population

The present study was conducted within a cohort of pregnant women in Budi Kemuliaan Hospital, Jakarta, Indonesia, a private municipal hospital that specialised in maternal and child health care, training (midwives), education and research. Recruitment of participants took place from July 2012 until October 2014. Muslim pregnant women who were paying antenatal care visits to the hospital were asked to participate and provided a written informed consent. Women were examined and interviewed by the midwives according to the standard clinical care and followed on the subsequent antenatal care visits until delivery. Pregnancies that ended with preterm birth were excluded since they intrinsically had less chance to be exposed to Ramadan and were more likely to result in lower-birth-weight babies.

### Ramadan and fasting exposure

The present study coincided with three Ramadan months, which occurred on 21 July–18 August 2012, 10 July–7 August 2013, and 29 June–27 July 2014. Pregnancies were classified as being exposed to Ramadan if the women's last menstrual period coincided with Ramadan or if Ramadan started after the women's last menstrual period and before delivery. All pregnancies that overlapped with Ramadan in any number of days were all classified as exposed. These exposed pregnancies were, further, classified according to the trimesters when the exposure occurred. They were consecutively classified as being exposed in the first, second and third trimester if Ramadan started between the 1st and 93rd day, the 94th to 186th day, and at 187th day or later of the pregnancy.

Maternal fasting behaviour was measured using a self-administered questionnaire in women who came to the hospital for antenatal care during Ramadan in 2013 (10 July 2013–7 August 2013). Women were classified as being exposed to Ramadan fasting if she had fasted for at least 1 d during Ramadan. Fasted women were also classified according to their trimester when they fasted.

### Dietary assessment

Assessment of women's dietary intakes was done by a nutrition officer using a single 24 h dietary recall, both in the fasting and non-fasting women and was repeated 1 month later. A 24 h dietary recall is a retrospective method of dietary assessment in which every individual is interviewed about his or her food and beverage consumption during the previous 1 d or 24 h. A computerised data analysis system (Nutrisurvey, 2007; Indonesian version) was used to convert food intake into nutrient intake based on the portion sizes, preparation of recipes and food tables. The system is a translation from a German nutrition software package (EBISpro), which has been adapted to the local food tables.

### Outcome measurement

Newborn birth weight was investigated in relation to both Ramadan exposure and maternal fasting. Birth weight was measured using a standard infant scale (Tanita).

### Data analysis

Maternal and babies’ characteristics were tabulated by Ramadan exposure and Ramadan fasting exposure separately, for both descriptive purposes and for initial evaluation for possible confounding. First, we compared the birth weight of newborns with Ramadan exposure during pregnancy with the unexposed. Second, within the Ramadan-exposed newborns, we examined the effect of maternal fasting on birth weight. A trimester-specific analysis was done to investigate if the effect differs according to the timing of exposure.

We used linear regression analysis to analyse the association between Ramadan exposure and maternal fasting on newborn birth weight. Univariable analyses were first performed and followed by multivariable analyses for adjustment for confounders. Potential confounders were secondhand smoking exposure, monthly family income and maternal education (as proxy for socio-economic status), pre-pregnancy BMI, gestational duration and parity. Secondhand smoking was used instead of active smoking because few (<1 %) of these pregnant women smoke. Family income was asked to the women as an estimate range. Women's education was categorised as low if the women had finished elementary or junior high school, middle if they had finished senior high school, or high if they attained education from university. Pre-pregnancy BMI was calculated as women's self-reported pre-pregnancy weight in kg divided by the square of height in metres. Women's weight was measured at each antenatal care visit and at the day of delivery using a standard weight scale (Camry). Gestational durations were measured as the difference between the last menstrual date and the date of delivery. Women's parity was classified into nulli- or multiparity. Results are expressed as β coefficients from linear regression with 95 % CI and corresponding *P* values. Statistical significance were considered to be a two-sided *P* < 0·05. All analyses were done with SPSS version 21.0 for Windows (SPSS Inc.).

## Results

**Fig. 1.**
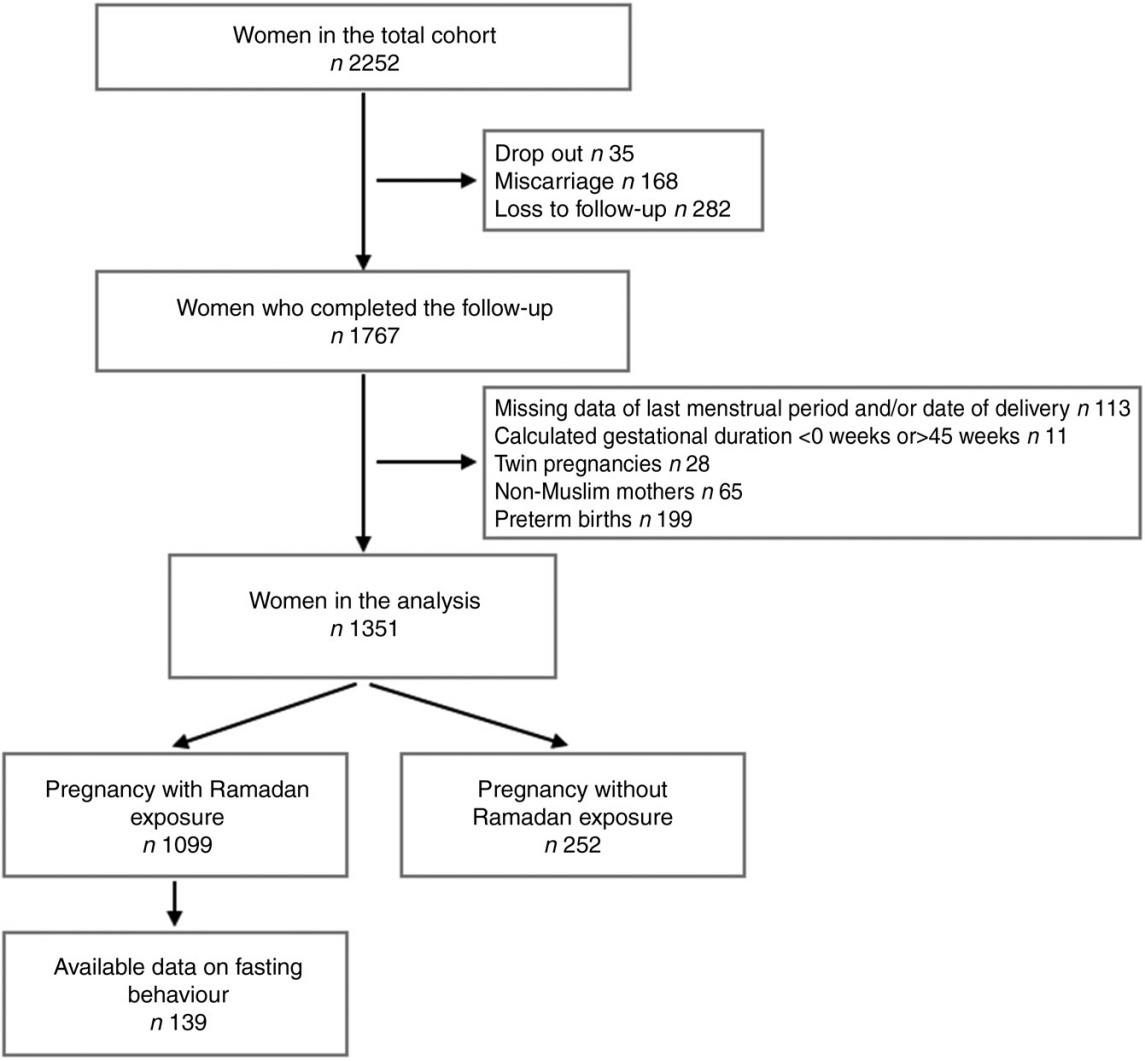
Overview of the study population.

The initial number of pregnant women in the total cohort was 2252. Thirty-five women withdrew their consent (drop outs), 168 women experienced miscarriage, and 282 women could not be contacted for their pregnancy outcomes (loss to follow-up). After excluding the women with missing data on their last menstrual period and/or date of delivery (*n* 113), miscoded gestational durations (*n* 11), twin pregnancies (*n* 28), non-Muslim mothers (*n* 65) and preterm births (*n* 199), the cohort included 1351 women for analysis. The flowchart of the study population is shown in Fig. 1.

The baseline characteristics of women based on their exposure to Ramadan during their pregnancy and their fasting status are shown in Table 1. The majority of women in this study were exposed to Ramadan during their pregnancy. Gestational duration was on average 4 d longer with Ramadan exposure. Women who were exposed to Ramadan had a slightly higher pre-pregnancy BMI as compared with the unexposed, although not statistically significant. The exposed and unexposed women were similar with respect to age, education, family income, secondhand smoking exposure, parity, pregnancy weight gain and babies’ sex.

**Table 1.** Baseline characteristics of women based on Ramadan exposure and their fasting status (Numbers of subjects and percentages; mean values with their standard errors; medians and interquartile ranges)

	Ramadan exposure					Ramadan fasting				
	Yes		No			Fasting		Non-fasting		
	*n*	%	*n*	%	*P*	*n*	%	*n*	%	*P*
Subjects	1099	81·3	252	18·7		110	79	29	21	
Maternal age (years)					0·38*					0·30*
Mean	28·2		28·6			28·8		27·4		
se	0·2		0·4			0·6		1·4		
Maternal pre-pregnancy BMI (kg/m^2^)†					0·09‡					0·03‡
Median	22·2		21·5			22·6		21·1		
Interquartile range	5·6		5·5			5·3		2·7		
Maternal weight gain (kg)					0·96*					<0·01*
Mean	12·3		12·2			11·6		17·6		
se	0·3		0·6			0·9		1·6		
Maternal education					0·13§					0·74ǁ
Low	204	18·6	53	21·0		10	9·1	4	13·8	
Middle	695	63·4	143	56·7		70	63·6	18	62·1	
High	197	18·0	56	22·2		30	27·3	7	24·1	
Monthly family income					0·31§					0·03ǁ
<1 million IDR	104	9·5	15	6·0		3	2·7	5	17·2	
1–2·5 million IDR	481	43·8	113	44·8		47	42·7	14	48·3	
2·5–5 million IDR	341	31·0	83	32·9		45	40·9	8	27·6	
> 5 million IDR	69	6·3	12	4·8		7	6·4	1	3·4	
Secondhand smoking exposure					0·57§					0·48ǁ
Yes, every day	234	47·0	61	50·8		12	37·5	4	57·1	
Yes, sometimes	66	13·3	12	10·0		4	12·5	0	0·0	
No	198	39·8	47	39·2		16	50·0	3	42·9	
Nulliparity	323	38·0	81	38·2	0·97§	46	42·6	13	44·8	0·83§
Gestational duration (d)†					<0·01‡					0·60‡
Median	278		274			276		277		
Interquartile range	14·0		12·0			11·5		12·5		
Baby's sex (boy)	562	51·3	134	53·2	0·59§	54	49·1	13	44·8	0·68§

IDR, Indonesian Rupiah.

* Independent-samples *t* test.

† Skewed data.

‡ Mann–Whitney test.

§ χ^2^ Test.

ǁ Fisher's exact test.

There were 139 women who came to the hospital between 10 July and 7 August 2013, from whom data about fasting behaviour were collected. As compared with Ramadan-exposed women who did not come to the hospital during the month, these women had higher gestational age (24·0 *v.* 19·0 weeks; *P* < 0·01). There were 110 (79 %) women who fasted to some extent during Ramadan and twenty-nine (21 %) women who did not fast at all. Among women who fasted, the median number of days fasted was 14 d. Fasting women had significantly higher pre-pregnancy BMI and less pregnancy weight gain than the non-fasting. There were also fewer fasting women in the lowest income category. The two groups were not different with respect to age, education, secondhand smoking exposure, parity, gestational duration and babies’ sex.

The Ramadan-unexposed pregnancies consisted of all pregnancies in the cohort that did not overlap with Ramadan and were taken as the reference group. Birth weight of Ramadan-exposed babies was on average 3107·5 (sd 545·7) g while that of unexposed was 3022·4 (sd 545·7) g. In Table 2, we show the association between Ramadan exposure during pregnancy and newborn birth weight. Newborn birth weight was not different between Ramadan-exposed and -unexposed groups, neither in crude nor in adjusted analysis. Similarly, Ramadan exposure in the first and second trimesters did not influence newborn birth weight. With exposure in the third trimester, birth weight was shown on average 73 g higher in the crude analysis and model 1, but this association was reduced in the fully adjusted analysis.

**Table 2.** Total and trimester-specific associations between Ramadan exposure during pregnancy and the birth weight of newborns† (Regression coefficients and 95 % confidence intervals)

		Crude model		Model 1‡		Model 2§	
	*n*	Regression coefficient	95 % CI	Regression coefficient	95 % CI	Regression coefficient	95 % CI
Ramadan unexposed	252	Reference		Reference		Reference	
Ramadan exposed (at any trimester)	1099	54·6	−3·4. 112·5	54·9	−3·1, 113·0	46·4	−12·4, 105·2
Ramadan exposed at first trimester	350	30·7	−37·8, 99·2	34·5	−34·1, 103·1	22·9	−46·2, 92·0
Ramadan exposed at second trimester	386	58·7	−8·4, 125·9	57·3	−9·9, 124·5	61·9	−6·5, 130·4
Ramadan exposed at third trimester	363	73·2*	5·2, 141·2	72·3*	4·1, 140·5	53·8	−15·5, 123·0

* *P* < 0·05.

† Linear regression coefficients indicated the difference in birth weight (g) compared with the reference category.

‡ Model 1 is adjusted for secondhand smoking exposure, family income categories and maternal education categories.

§ Model 2 is adjusted for as model 1 and for pre-pregnancy BMI, gestational duration and parity.

**Fig. 2.**
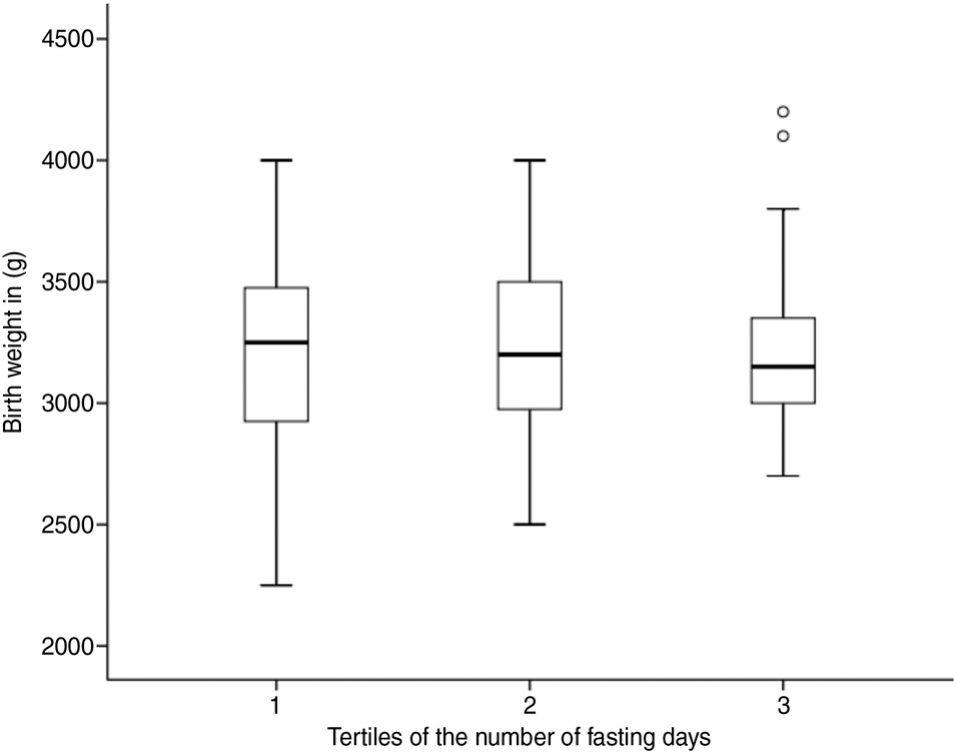
Boxplot showing the relationship between tertiles of fasting days and birth weight. Tertile 1 (*n* 33), tertile 2 (*n* 40) and tertile 3 (*n* 35). The horizontal lines represent medians, the boxes interquartile ranges, the whiskers are minima and maxima and the circles are outliers.

Within women who were exposed to Ramadan during their pregnancy, comparison of their newborns' birth weight was made according to the fasting status. Average birth weight of babies who were exposed to Ramadan fasting during pregnancy was 3201·5 (sd 377·6) g and 3190·6 (sd 606·7) g in babies who were not. As shown in Table 3, birth weight of newborns of the fasted women was not significantly different from those who did not fast, both in the crude and adjusted analysis. Although not statistically significant, trimester-specific analysis shows a trend towards reduced birth weight with fasting, especially in women who fasted in the second and third trimesters. Furthermore, within fasted women, we also explored if birth weight differed according to fasting intensity. Fig. 2 shows a boxplot that illustrates the association between tertiles of fasting days with newborn birth weight. The first tertile of fasting days consists of women who fasted for 1 to 8 d, while the second and third tertiles include women who fasted for 10 to 23 and 24 to 30 d.

**Table 3.** Total and trimester-specific associations between maternal fasting during pregnancy and the birth weight of newborns* (Regression coefficients and 95 % confidence intervals)

		Crude model		Model 1†		Model 2‡	
	*n*	Regression coefficient	95 % CI	Regression coefficient	95 % CI	Regression coefficient	95 % CI
Did not fast	29	Reference		Reference		Reference	
Fasted (at any trimester)	110	−63·7	−230·5, 103·0	−62·3	−241·2, 116·6	−72·1	−257·6, 113·5
Fasted at first trimester	23	−23·4	−246·8, 200·1	−17·1	−274·6, 240·4	−0·7	−260·8, 259·4
Fasted at second trimester	47	−35·3	−224·2, 153·7	−31·5	−233·2, 170·2	−38·1	−244·6, 168·3
Fasted at third trimester	40	−121·8	−318·0, 74·4	−116·2	−326·4, 94·1	−144·1	−360·2, 72·0

* Linear regression coefficients indicated the difference in birth weight (g) compared with the reference category.

† Model 1 is adjusted for secondhand smoking exposure, family income categories and maternal education categories.

‡ Model 2 is adjusted for as model 1 and for pre-pregnancy BMI, gestational duration and parity.

During Ramadan, we performed a 24 h dietary recall in ninety-six women. There were forty-two women who were fasting and fifty-four women who were not fasting at the day of the interview. The median of gestational age was not different between the fasting and non-fasting women (33 *v.* 30 weeks; *P*  =  0·46). Table 4 shows the comparison of intake between these two groups. Women who were fasting reported significantly lower percentage of energy intakes from protein than the non-fasting. They reported lower total energy and protein intake, although borderline statistically significant. They also, in general, reported lower intakes of carbohydrates, water, vitamin A, Na and K, although none was statistically significant. There was no difference in consumption of fat, dietary fibre, PUFA, cholesterol, carotene, vitamin E, vitamin B_1_, vitamin B_2_, vitamin B_6_, folic acid, vitamin C, Ca, Mg, P, Fe and Zn, as well as percentage of energy from fat and carbohydrates.

**Table 4.** Maternal dietary intake on one Ramadan day based on fasting status* (Mean values with their standard errors; medians and interquartile ranges)

	Fasting (*n* 42)		Non-fasting (*n* 54)		
	Mean	se	Mean	se	*P*
Total energy					0·07
kcal	1646·1	60·5	1831·7	80·8	
kJ	6887·3	253·1	7663·8	338·1	
Water (g)	1870·2	92·5	1940·1	114·3	0·64
Protein (g)†	50·9	25·7	63·6	39·13	0·07
Fat (g)†	55·5	48·3	58·1	48·0	0·53
Carbohydrates (g)	215·2	9·3	232·8	9·9	0·21
Dietary fibre (g)†	10·4	7·2	9·8	5·7	0·62
PUFA (g)†	11·2	15·6	8·9	15·0	0·55
Cholesterol (mg)†	227·5	266·3	217·4	264·5	0·30
Vitamin A (mg)†	1076·2	1244·2	1377·0	2093·9	0·16
Carotene (mg)†	0·0	245·4	0·0	398·2	0·47
Vitamin E (mg)†	0·0	3·6	0·0	2·4	0·94
Vitamin B_1_ (mg)†	0·6	0·6	0·7	0·6	0·81
Vitamin B_2_ (mg)†	0·9	0·6	1·1	0·92	0·63
Vitamin B_6_ (mg)†	1·2	0·9	1·2	1·0	0·66
Folic acid (μg)†	0·0	339·4	0·8	260·8	0·67
Vitamin C (mg)†	63·6	87·5	76·0	114·6	0·60
Na (mg)†	384·2	627·8	632·3	911·0	0·14
K (mg)	1793·2	142·6	1906·9	104·3	0·51
Ca (mg)†	588·6	507·8	571·4	569·9	0·96
Mg (mg)	255·6	17·4	264·5	15·5	0·70
P (mg)†	843·2	472·4	937·7	865·7	0·45
Fe (mg)†	10·4	13·1	11·5	12·4	0·98
Zn (mg)	7·2	0·4	8·3	0·5	0·14
Protein (% TE)	12·9	0·6	15·0	0·4	<0·01
Fat (% TE)	29·3	2·5	29·8	1·9	0·86
Carbohydrates (% TE)	52·2	1·3	51·6	1·1	0·73

% TE, percentage of total energy.

* Results are based on estimation using 24 h nutrition recall. *P* values are based on Student's *t* test or the Mann–Whitney *U* test in the case of skewed data.

† Skewed data. Medians and interquartile ranges.

In Table 5, we compared dietary intake of the fasting women during Ramadan with their intake 1 month later. There were fifteen women who could be re-interviewed for this purpose. During Ramadan, fasting women reported significantly less consumption of water, fat and vitamin A, and had a lower percentage of energy intake from carbohydrates. Water and fat intakes were significantly lower than 1 month later by on average 570 and 16 g while carbohydrate intake was approximately 50 g lower. Intakes of total energy, carbohydrates, vitamin C, Na, Ca and P, as well as percentage of energy from protein and fat were not significantly different.

**Table 5.** Maternal dietary intake on one Ramadan day as compared with 1 month after Ramadan within fasting women* (Mean values with their standard errors; medians and interquartile ranges)

	During Ramadan (*n* 15)		After Ramadan (*n* 15)		
	Mean	se	Mean	se	*P*
Total energy					0·22
kcal	1527·3	103·2	1744·1	165·3	
kJ	6390·2	431·8	7297·3	691·6	
Water (g)	1686·5	110·9	2255·3	152·6	<0·01
Protein (g)	52·5	5·0	54·8	5·0	0·69
Fat (g)†	42·0	63·3	58·1	34·2	0·02
Carbohydrates (g)	196·3	16·4	246·9	26·0	0·07
Dietary fibre (g)	10·4	0·9	10·1	1·2	0·80
PUFA (g)†	11·6	12·0	7·7	8·1	0·41
Cholesterol (mg)	234·7	45·3	228·8	40·5	0·93
Vitamin A (mg)†	1049·8	1224·3	2011·5	1581·7	0·02
Carotene (mg)†	0·0	356·3	0·0	0·0	0·06
Vitamin E (mg)†	0·0	2·4	0·0	0·3	0·51
Vitamin B_1_ (mg)†	0·6	0·4	0·5	0·4	0·87
Vitamin B_2_ (mg)	0·8	0·10	0·8	0·14	0·82
Vitamin B_6_ (mg)	1·2	0·14	1·2	0·16	0·97
Folic acid (μg)†	0·0	210·0	0·0	6·5	0·95
Vitamin C (mg)	62·4	12·0	78·7	16·2	0·40
Na (mg)†	383·9	644·9	285·9	906·8	0·78
K (mg)	1583·4	153·7	1589·0	191·1	0·98
Ca (mg)	485·9	71·7	378·9	71·5	0·26
Mg (mg)	240·1	25·6	222·6	25·2	0·59
P (mg)†	835·2	448·4	646·5	321·4	0·39
Fe (mg)†	8·2	12·4	8·0	4·4	0·23
Zn (mg)	6·3	0·6	6·5	0·6	0·81
Protein (% TE)	13·9	1·1	13·0	0·7	0·49
Fat (% TE)†	29·8	38·1	29·5	7·9	0·09
Carbohydrates (% TE)	50·8	2·3	56·4	1·5	0·01

% TE, percentage of total energy.

* Results are based on estimation using 24 h nutrition recall. *P* values are based on the paired-samples *t* test or Wilcoxon signed-ranks test in the case of skewed data.

† Skewed data. Medians and interquartile ranges.

In Table 6, we compared the dietary intake of the non-fasting women on 1 d of Ramadan with 1 month later. There were twenty-seven women who were accessible for this analysis. As compared with 1 month later, these women reported a consumption of significantly less water and had a lower percentage of energy intakes from protein. Intakes of all other nutrients were similar during Ramadan as compared with 1 month later.

**Table 6.** Maternal dietary intake on one Ramadan day as compared with 1 month later in women who were not fasting* (Mean values with their standard errors; medians and interquartile ranges)

	During Ramadan (*n* 27)		After Ramadan (*n* 27)		
	Mean	se	Mean	se	*P*
Total energy					0·56
kcal	1888·3	132·7	1986·9	112·4	
kJ	7900·6	555·2	8313·2	470·3	
Water (g)	1965·7	179·1	2462·8	90·6	<0·01
Protein (g)†	68·7	56·7	58·9	26·6	0·26
Fat (g)	62·2	7·8	69·2	4·7	0·44
Carbohydrates (g)	245·6	16·5	281·7	18·2	0·10
Dietary fibre (g)†	9·0	7·7	9·8	8·0	0·48
PUFA (g)†	9·6	16·6	11·0	10·2	0·98
Cholesterol (mg)†	234·4	274·3	247·6	203·8	0·55
Vitamin A (mg)†	1743·9	2573·6	2442·4	1541·6	0·52
Carotene (mg)†	0·0	8·0	0·0	0·0	<0·01
Vitamin E (mg)†	0·5	3·6	0·1	3·1	0·79
Vitamin B_1_ (mg)†	0·8	0·8	0·9	0·7	0·87
Vitamin B_2_ (mg)†	1·2	1·2	1·1	0·6	0·51
Vitamin B_6_ (mg)†	1·6	1·1	1·7	1·2	0·80
Folic acid (μg)†	2·8	351·2	4·0	350·0	0·82
Vitamin C (mg)†	58·7	118·3	73·7	83·5	0·60
Na (mg)†	560·4	580·0	533·8	672·7	0·85
K (mg)	1978·2	165·3	1839·3	164·3	0·45
Ca (mg)†	530·6	659·7	620·6	753·1	0·88
Mg (mg)	282·7	25·3	279·2	20·0	0·91
P (mg)†	1151·9	893·0	958·9	498·5	0·14
Fe (mg)†	14·3	12·7	10·4	10·3	0·06
Zn (mg)†	9·7	8·2	7·8	4·7	0·34
Protein (% TE)	15·4	0·6	13·0	0·6	0·01
Fat (% TE)†	28·2	11·9	30·7	8·9	0·41
Carbohydrates (% TE)	52·8	1·8	56·2	1·5	0·14

% TE, percentage of total energy.

* Results are based on estimation using 24 h nutrition recall. *P* values are based on the paired-samples *t* test or Wilcoxon signed-ranks test in the case of skewed data.

† Skewed data. Medians and interquartile ranges.

## Discussion

Approximately 81 % of all women in our study had Ramadan exposure at any time during their pregnancies and about 79 % of them fasted to some extent during the month. Ramadan exposure during pregnancy was not significantly associated with newborn birth weight. Furthermore, within the Ramadan exposed, babies’ birth weight of the fasted women was not significantly different from that of the babies of non-fasted women. In the present study, we showed that fasted women reported a significantly lower intake of total energy, macronutrients and water as compared with women who did not fast. Women's intakes were also generally lower during Ramadan as compared with 1 month later, both in the fasted and non-fasted group.

To the best of our knowledge, we are the first to study the effect of Ramadan during pregnancy on birth weight by looking both on the effects of Ramadan overlap and maternal fasting behaviour. We are also among the first to evaluate women's dietary intake during and after Ramadan, both in the fasting and non-fasting group.

There were some limitations of the present study. First, interviews about women's fasting behaviour were limited only to women who came to the hospital during the period of data collection. The relatively small number of women analysed in the association between fasting and babies’ birth weight could limit statistical power, thus requiring cautious interpretation of the findings. Women with available data on fasting behaviour were at a later stage of gestation as compared with women without. Furthermore, women with available data on nutritional intake were also at a later gestational age (24 *v.* 19 weeks; *P* < 0·01) and had lower pre-pregnancy BMI (22·2 *v.* 23·2 kg/m^2^; *P*  =  0·05) as compared with women without nutritional data. The average pregnancy weight gain was not different in women with available nutrition data as compared with those without (12·9 *v.* 12·3 kg, *P*  =  0·88). The difference in gestational age could be caused by the fact that women at later gestational age had more visits to the hospital, while we can only speculate about the difference in BMI. Gestational diabetes mellitus, notably, could confound the relationship between Ramadan fasting and newborn birth weight; however, we could not adjust for it in the analysis due to the absence of gestational diabetes mellitus screening in our cohort. Furthermore, the use of single 24 h dietary recall as nutritional assessment may give some disadvantages since it relies on subjects' memory, cooperation and communication ability, and it may not have captured the day-to-day variation in nutrient intake.

We are aware of the possibility of reverse causation, where babies with longer gestational duration (and therefore higher birth weight) had higher chance to be exposed to Ramadan. We dealt with it by restricting the analyses to the full-term babies. Although we had excluded the preterm babies, we still found that pregnancies with Ramadan overlap were on average 4 d longer than those without (Table 1). Thus, gestational length was an important potential confounder to be adjusted for in our main analysis.

Several other possible confounders were included in our model as those variables were found to be associated both with the exposure and with the outcome. Secondhand smoking exposure, lower family income and low maternal education were slightly more common in the non-fasting women. Fasting women also had a significantly higher pre-pregnancy BMI and lower gestational weight gain than the non-fasting. These women may feel healthier and therefore more prone to fast. In the previous studies, several maternal characteristics have been shown to predict maternal fasting during Ramadan, such as pre-pregnancy BMI, parity, maternal age and socio-economic status^(^^17^^,^^32^^,^^34^^,^^35^^)^.

Our finding of no significant difference in birth weight of babies with or without Ramadan overlap during pregnancy is in accordance with several previous studies^(^^15^^,^^27^^,^^36^^)^. These studies were, however, mostly based on registry data with limited information on women's religion and might therefore have included non-Muslim women in their analysis. Our finding of no significant difference in babies’ birth weight in the fasting women as compared with the non-fasting women is also in line with several earlier studies^(^^16^^–^^18^^,^^26^^,^^29^^,^^30^^)^, although different from our previous study among pregnant Muslim women in Amsterdam (The Netherlands). In the previous study, a lower birth weight was found with maternal fasting in the first trimester^(^^32^^)^. The difference in the finding could be attributed to the contrast in cultural and dietary habits of these women, who were mostly of Turkish and Moroccan background. Variation in the climate, duration and the number of fasting days could also influence the degree of exposure.

Our dietary assessments showed that women who were fasting during Ramadan in general reported lower percentage of total energy intake from protein, intake of total energy and macronutrients, as well as water, vitamin A, Na and K as compared with the non-fasting women. The differences in women's intakes were unlikely to be caused by the difference in their gestational age since women in both groups had similar gestational age at the interview. This result is in accordance with findings from previous studies on dietary intake of pregnant women during Ramadan which found significantly lower energy intake among women who fasted as compared with those who did not^(^^22^^)^, and lower energy intake during Ramadan as compared with outside Ramadan^(^^37^^)^. In contrast, our result is different from a study conducted among Saudi families which reported weight gains due to higher consumption of carbohydrates and fat-rich food during Ramadan^(^^9^^)^.

Ramadan also appeared to influence dietary intakes of women both in the fasting and non-fasting groups. It was shown by lower intakes of total energy, carbohydrates, fat and water during Ramadan as compared with intakes of the same women 1 month later, both in the fasting and non-fasting groups. More substantial changes of the intake during Ramadan as compared with 1 month later, however, were evident among fasting women. We suggest that in the non-fasting women, dietary habits were also influenced by their fasting families, as seen by the reduction of the frequency and/or the portion size of women's meals. Furthermore, as compared with the Indonesian estimated average requirement for pregnant women, we found that the mean intakes of protein and fat of women in our study consistently exceeded the recommendation, while carbohydrate intakes were generally lower than recommended. The estimated average requirements for protein, fat and carbohydrate are 43–52, 40–42 and 270–285 g, respectively^(^^38^^,^^39^^)^.

Despite the nutrition restriction during Ramadan, a significant reduction of (secondhand) smoke exposure is expected since Muslims are not allowed to smoke during daylight^(^^40^^,^^41^^)^. This temporary change may, to some extent, influence the birth weight of Ramadan-exposed babies. Furthermore, Ramadan is followed afterwards by several days of *Eid* celebration. During this celebration, people gather with family and friends and usually have meals with high fat and sugar contents. *Eid* celebration, which is less likely to be experienced by women who did not have Ramadan in their pregnancy, may enable the exposed pregnant women to compensate.

Our study showed that women who were exposed to Ramadan and did not fast had the highest weight gain during pregnancy (17·6 (se 1·6) kg), as compared with women who fasted (11·6 (se 0·9) kg) and Ramadan-unexposed women (12·2 (se 0·6) kg). This finding is consistent with several studies^(^^16^^,^^22^^,^^23^^,^^42^^)^ which reported significantly lower weight gain in women who fasted during pregnancy as compared with women who did not. Furthermore, women who were exposed to Ramadan and did not fast might have had the highest fat or sugar intake, through the consumption of seasonal meals during and shortly after Ramadan.

In conclusion, in Indonesian Muslim women lifestyle changes that occur with Ramadan are associated with lower reported energy intake. Ramadan overlap during pregnancy was not significantly associated with offspring birth weight while we cannot conclude on the effect of fasting on birth weight due to limited statistical power. Further studies with larger sample size focusing on other pregnancy outcomes and the offspring's long-term health are needed.
